# Influence of Authentic Leadership on Employees’ Taking Charge Behavior: The Roles of Subordinates’ *Moqi* and Perspective Taking

**DOI:** 10.3389/fpsyg.2021.626877

**Published:** 2021-05-25

**Authors:** Qiuxiang Wen, Ruhong Liu, Jing Long

**Affiliations:** School of Business, Nanjing University, Nanjing, China

**Keywords:** authentic leadership, subordinates’ *moqi*, perspective taking, taking charge behavior, role identity

## Abstract

How to motivate employees to break through the role constraints and show more initiative determines the success or failure of a company’s future development. Taking charge behavior refers to the behavior where individuals influence the change of organizational function through voluntary and constructive efforts, which is a challenging organizational citizenship behavior. This study investigates the underlying mechanism and boundary condition of authentic leadership (AL) on employees’ taking charge behavior based on the role identity theory and literature concerning perspective taking. Matched data were collected from a multi-source sample that included 146 direct supervisors and 328 subordinates in mainland, China. The empirical results indicate that AL has a positive influence on the employees’ taking charge behavior, and subordinates’ *moqi* mediates the relationship between them. In addition, the employees’ perspective taking positively moderated the positive relationship between AL and subordinates’ *moqi*, as well as the mediating effect of subordinates’ *moqi* in the relationship between AL and employees’ taking charge behavior. Compared with the low levels of perspective taking, high levels of that made the influence of AL on subordinates’ *moqi* stronger, so is the whole indirect effect. This study is the first to explore the influencing mechanism of AL on employees’ taking charge behavior from the perspective of the role identity theory, thereby enriching the relevant studies and providing practical insights for organizational leaders regarding on how to foster employees to take charge.

## Introduction

With the extensive application and rapid development of the Internet and artificial intelligence technology in various industries, the survival and development of enterprises have encountered more technical challenges in the recent year. How to further promote technological innovation has evidently become an issue for enterprises. Meanwhile, in today’s highly dynamic and uncertain environment, especially when enterprises face extremely unpredictable events such as the COVID 19 pandemic, enterprise managers increasingly hope that employees will no longer be confined to narrow, prescribed tasks, and can actively engage in a wider range of tasks (Dysvik et al., 2016; Zhan et al., 2020), which is particularly important for the survival and development of enterprises. Hence, employees bring a continuous vitality to an organization’s development through proactive behaviors, such as speaking up (Wen et al., 2020), organizational citizenship (McAllister et al., 2007), and innovative behaviors (Janssen, 2005). Although these extra-role activities are important, they are not sufficient to ensure the sustainability of an organization, and organizations also need employees who are willing to challenge the present operating conditions to bring constructive changes (Morrison and Phelps, 1999). Until recently, as a kind of a positive transformative behavior, taking charge has attracted much attention (Parker et al., 2010). Taking charge refers to the “voluntary and constructive efforts, by individual employees, to effect an organizationally functional change with respect to how work is executed within the contexts of their jobs, work units, or organizations” (Morrison and Phelps, 1999). Unlike other initiatives, taking charge behavior lies in improvement and change (Love and Dustin, 2014), which is often highly challenging and risky (Parker et al., 2010), and further requires employees to have the ability and willingness to change. Accordingly, organizational leaders must pay attention to the performance of employee’s taking charge behavior for the sustainable development of organizations (Lin and Zhao, 2016). As such, our research attempts to explore the factors that compel employees to take charge at work, thus effectively stimulating them to take charge actively.

Reviewing the relevant literature on taking charge, the majority of researches mainly explored the influencing factors and mechanisms from two paths, namely, individual characteristics and situational factors. The former pays additional attention to an individual’s personality (Fuller et al., 2012; Kim and Liu, 2017), ability (Hu and Ji, 2018), motivation (Cai et al., 2018), and values (Seppala et al., 2012), while the latter focuses on the human resource management practices (Yang et al., 2019), team atmosphere (Müceldili and Erdil, 2016), and leadership behaviors (Li et al., 2016; Xie and Xi, 2018; Zhan et al., 2020). For example, Fuller et al. (2012) and Kim and Liu (2017) studied the influence of a proactive personality on employee’s taking charge behavior. Many researchers also emphasize the vital role of leaders in motivating employees to take charge, such as an empowering (Li et al., 2016), humble (Xie and Xi, 2018), and coaching leadership (Zhan et al., 2020). The leadership factor has attracted much attention from scholars. As one of the new leadership theories, authentic leadership (AL) is characterized by honesty, integrity, internalized morality, deep understanding of self-worth (Walumbwa et al., 2008), and advocating transparent relationships with employees to obtain the employees’ psychological identity (Wen et al., 2020). In addition, previous studies showed that AL has a positive influence on the employees’ individual behavior and attitude and implementation of organizational goals (Peterson et al., 2012; Hannah et al., 2014; Wang and Zhang, 2019). Therefore, this study infers that AL can promote employee’s taking charge behavior. In the existing research, only scholar Lin and Zhao (2016) made a preliminary analysis of the relationship between them, which is far from enough and remains much space for further exploration in terms of the mechanisms and boundary conditions through which AL influences employee’s taking charge behavior.

At present, scholars generally take the social exchange theory as the theoretical foundation in their studies and explain the influencing mechanism of AL on employee’s taking charge behavior through the leader–member exchange relationship (Rego et al., 2013). However, this theoretical explanation mechanism cannot fully reflect the characteristics and essence of AL. Specifically, compared with other leadership behaviors, the biggest advantage of AL is that it reflects the characteristics of sincerity, high morality, and balanced information processing in the interaction with employees, and then, influence them. In view of this, this article attempts to explore the mechanism of AL on employee’s taking charge behavior from a new perspective—the role identity theory (Stryker, 1980, 1987; Burke, 1991)—and regards the influence as a process to promote the employees and supervisors to reach a fit state. As a reflection of their understanding of the supervisors’ work expectations, requirements, and behavioral intentions (Zheng et al., 2017b), to a certain extent, subordinates’ *moqi* has a positive influence on the individuals’ work attitude and behavior (Xing and Liu, 2015). Therefore, we choose subordinates’ *moqi* as our mediator, through which AL increases employee’s taking charge behavior. More specifically, the strong self-awareness and morality of AL lead an effective “action roadmap” for the employees to reach a *moqi* state among them, thereby promoting the employees to take charge, which reflects the process of the employees’ role identity of leaders. As such, this study provides a more appropriate perspective for understanding the nature of AL and its influence on employee’s taking charge behavior.

According to Stryker’s role identity theory, the process of an individual’s role identity will also be affected by his or her factors. For example, perspective taking reflects the ability of an individual to consciously focus on understanding another person’s viewpoint and making cognitive and emotional reactions (Parker et al., 2008), which will affect subordinates’ *moqi* with their supervisors. Specifically, subordinates with a high perspective taking will invest in more cognitive resources to likely understand the leaders’ intention or idea (Gilin et al., 2013), promote subordinates to effectively interpret the characteristics or clues presented by AL, and finally, reach a *moqi* state. By contrast, subordinates with a low perspective taking may prefer interpreting the leaders’ behavior from their own perspective to investing in enough cognitive resources. Hence, being too subjective may cause a cognitive bias (Yaniv and Choshen, 2012), which is not conducive to the cultivation of a *moqi* between the subordinates and supervisors, and then, decreases taking charge. Therefore, we propose perspective taking as the boundary condition between AL and employee’s taking charge behavior.

Our research makes several contributions to extant literature concerning AL and taking charge behavior. First, this study reveals the positive effect of AL on taking charge behavior, and deepens the theoretical understanding of AL. Incorporating employee’s taking charge behavior into the research on influence of AL expands the influencing effect research of AL as a new type of leadership behavior. Moreover, this case enriches the theoretical research of exploring the antecedents of taking charge from the leader factor. Second, we explore the influencing mechanism of AL on employee’s taking charge behavior for the first time based on the perspective of the role identity theory. Third, this study expands the boundary conditions for the influence of AL by exploring the moderating role of the employees’ perspective taking, which is highly conducive to comprehensively reflect the influence of the leadership style on the employees’ behavior.

## Theoretical Background and Hypotheses

### Authentic Leadership

The “authenticity” concept in authentic leadership (AL) is derived from the Greek philosophy of “loyal to oneself” (Steffens et al., 2016). This concept refers to the personal experience to understand oneself, whether it refers to thoughts, emotions, needs, preferences, or beliefs. The process of capturing means that people should act according to their true selves and express themselves in a way that is consistent with their inner thoughts and feelings (Lopez et al., 2002; Hirst et al., 2016). On the basis of summarizing positive psychology and positive organizational behavior, Luthans et al. (2003) defined AL, for the first time, as “a leadership process rooted in a highly developed organizational context and positive psychological capital” in which leaders and subordinates can gain a higher self-awareness and demonstrate positive behaviors of self-regulation. Likewise, Shamir and Eilam (2005) believed that authentic leaders tend to have a high level of self-confidence, a clear self-awareness, a high degree of alignment with their goals, and behaviors that express self-will.

During the early times, AL was conceptualized in different ways, and gradually developed into a multi-dimensional mature construct as the theory evolved. For instance, Ilies et al. (2005) proposed the AL construct with four dimensions, namely, self-awareness, unbiased information processing, real behavior, and authentic relationship orientation. Walumbwa et al. (2008) also defined AL from multiple dimensions, “AL can promote the positive psychological capital of employees and create a positive moral atmosphere.” Authentic leaders have a high level of self-awareness and internalized ethics, can balance information processing, maintain transparency in relationships with subordinates at work, and facilitate positive self-growth of employees. We follow Walumbwa’s conceptualization of AL and focus on its four dimensions: ➀ Self-awareness reflects positive self-concepts, enabling leaders to accurately assess their own internal characteristics (values, strengths, and weaknesses) and can become aware of their influence on their subordinates; ➁ Internalized morality reflects the internalization and integrity of self-regulation, that is, to what extent the leaders are guided by their internal moral standards and values, and even in the face of external pressure, they still insist on the behavior that is consistent with their internal values; ➂ Relational transparency refers to the fact that leaders show their true selves when getting along with others, and establish positive trust relationships with their subordinates by sharing information and expressing true feelings; ➃ Balanced processing means that leaders can collect information, analyze, and make decisions objectively and without prejudice, and they provide employees with the opportunity to express their own ideas and opinions, regardless whether the opinions challenge their beliefs and values.

### Authentic Leadership and Employees’ Taking Charge Behavior

According to Morrison and Phelps, the survival of modern enterprises requires employees to be highly committed to the extra-role behaviors that they transcend the tasks described in the job to perform and achieve organizational goals. Taking charge behavior, as an important extra-role behavior, is defined as a spontaneous and constructive behavior of organization members, which aimed to change and influence the work behavior of the organization (Morrison and Phelps, 1999). The common denominator of employee’s taking charge behavior and other extra-role behaviors (e.g., voice, organizational citizenship, and innovation behavior) is voluntary. What differs from others is that the nature of taking charge is change- and improvement-oriented, that is, the *status quo* is oriented to change rather than maintain (Parker and Collins, 2010). Hence, taking charge behavior is a risky and challenging proactive work behavior that requires the employees to have a strong sense of psychological security to take charge (Yang et al., 2019). In addition, Cai et al. classified the psychological mechanism that motivates the employees to take charge as “can do,” “reason to,” and “energized to.” Therefore, employee’s taking charge behavior often needs organizational support resources, intrinsic motivation, and a strong sense of work security (Cai et al., 2019).

Previous studies have confirmed the influence of AL on the proactive work behaviors of organization members (Walumbwa et al., 2010; Searle and Barbuto, 2013). The authentic leaders in the four aspects of self-awareness, relationship transparency, internalized morality, and balanced processing play a positive role in promoting employee’s taking charge behavior. First, authentic leaders help to inspire the employees’ willingness to take charge. Self-awareness emphasizes that leaders can objectively understand themselves and learn about the abilities, strengths, and contributions of their subordinates. These managerial styles and practices help the employees have a clear understanding of their responsibilities and obligations beyond the job requirements. Additionally, such styles can encourage the employees to take the initiative to participate in a wider range of work roles and enhance their ability to take charge beyond their roles. In turn, the recognition and feedback of employee’s taking charge behavior makes the employees greatly believe in their own capabilities and become willing to believe that taking charge can bring positive results to the organization and themselves. Relationship transparency shows that leaders will establish candid, open, and equal interpersonal relationships with their subordinates, shorten the distance between the two parties, actively share information, and encourage their subordinates to speak their true thoughts. Maintaining a good relationship with their subordinates, affirming, and appreciating their advantages can promote the employees’ job satisfaction and organizational recognition, which can motivate the employees to help other colleagues. Second, authentic leaders promote employee’s taking charge behavior by providing organizational resources, such as work information and psychological support. Leaders with balanced information processing characteristics maintain maximum objectivity and fairness to the information provided by members of different organizations. Moreover, they are open and inclusive to new ideas and feedback. According to the research results of Morrison and Phelps (1999), the openness shown by the management has a positive effect on employee’s taking charge behavior. Based on this, the balanced information processing method of AL can facilitate the employees to take charge. Finally, more “responsibility” is often accompanied by more “risk” for the taking charge behavior is an extra-role action. However, the internalized morality dimension of AL can reduce the risk of taking charge (Borgersen et al., 2014), provide employees with a sufficient work security, and then, promote their taking charge behavior. Hence, we propose the following hypothesis:

Hypothesis 1: *AL positively influences employee’s taking charge behavior.*

### The Mediating Role of Subordinates’ *Moqi*

*Moqi* reflects a state between two parties, whereby one party implies something and the other senses and understands it tacitly. In the workplace context, *moqi* can exist across supervisors and subordinates, members of a team, employees with external customers, and countless other relationships (Zheng et al., 2017b). Similar to Zheng, we mainly focus on the supervisor–subordinate relationship and settle in a subordinate-centric view (i.e., subordinates’ *moqi*). In addition, we believe that *moqi* reflects the degree to which subordinates can implicitly feel and understand the work expectations, requirements, plans, and behavior intentions of their supervisors.

AL is a process that combines the positive psychological abilities of the leader with the highly developed organizational environment. On the one hand, authentic leaders have a strong self-awareness and noble moral sentiments, show self-regulated positive behaviors, can make better use of their own speech and behavior, and cultivate AL with employees by strengthening their own and moral values. To exert their role as role models, they never manipulate their subordinates, always focus on the advantages and career development of their employees, and take the initiative to seek benefits for their employees, so that their subordinates perceive a higher level of supervisors and organizational trust (Walumbwa et al., 2008). At this point, if individuals believe that a certain state or result is necessary to reach, they will set goals and create an “action roadmap” to achieve a better match between themselves and the internal working environment, which is consistent with the proactive motivation model proposed by Parker et al. (2010). Subordinates who have *moqi* with authentic leaders can accurately judge the leader’s intention, greatly complete tasks, and reach or exceed the leader’s expectations, which contribute to obtaining a more positive evaluation and expectations from the leaders (Zheng et al., 2017b). Therefore, the moment the subordinates perceive the AL of their leaders, they will regard them as a role model which has a key influence on their career development, and they increasingly hope to reach a *moqi* state with their leaders. On the other hand, authentic leaders actively share knowledge and information, advocate to establish a harmonious and trusting relationship with their subordinates, and encourage their subordinates to express their views and values. These factors are conducive to the formation of a harmonious and equal organizational atmosphere and enhance the frequent communication between supervisors and subordinates, thus, promoting the cultivation of *moqi* between them. As such, we believe that AL can promote the cultivation of subordinates’ *moqi*.

A good cooperative interaction due to the common cognition that reduces the psychological distance between the two parties, that is, the establishment of a higher *moqi* can bring more positive emotional experiences to the subordinates in the transformation work (Zheng et al., 2017b). First, subordinates’ moqi is a dynamic process that requires the subordinates to actively cultivate. Frequent communication in the cultivation process allows the leaders to allocate additional attention and resources to the subordinates with *moqi*, and makes the subordinates believe that they are needed and recognized, so the subordinates are more willing to implement proactive actions. Second, according to the role identity theory, when the role bearer understands his own role expectations, he will play the corresponding role image according to the established role norms and show the corresponding role behavior (Grant et al., 2011). In the workplace, the *moqi* between subordinates and leaders allows the subordinates to quickly understand the self-evident expectations and goals of the leaders, reducing the uncertainty in the interaction between them, thus, the subordinates can easily meet the leaders’ expectations. In obtaining the approval and support of the leaders by meeting their expectations, it not only helps the subordinates develop a sense of psychological security and reduce the risk of a proactive behavior, but also helps them to obtain more psychological support, work information, and other organizational resources, which inspires their willingness and sense of responsibility to take charge (Matta et al., 2015), so as to stimulate more taking charge behavior from the subordinates. Conversely, a lower level of *moqi* between subordinates and supervisors is not conducive to a mutual understanding. Moreover, the process of work interaction is more likely to cause disagreements between the two parties, and result in negative emotions felt by the subordinates. Meanwhile, supervisors will weaken their help and support due to the subordinates’ negative attitude. This case will lead to the lack of a necessary resource for the employees’ taking charge, and their willingness to take charge will decrease, thus reducing employee’s taking charge behavior (Ma et al., 2020).

According to the above discussion, AL can significantly improve subordinates’ moqi, and subordinates’ moqi will further encourage them to engage in a taking charge behavior. That is, AL will indirectly promote employee’s taking charge behavior via subordinates’ moqi. In light of the mentioned explanations, we propose the following hypotheses:

Hypothesis 2: *AL positively influences subordinates’ moqi.*
Hypothesis 3: *Subordinates’ Moqi mediates the relationship between AL and employees’ taking charge behavior.*

### The Moderating Role of Perspective Taking

According to a previous research, the effective unity of organizational situation and personal characteristics is a powerful driving force for the employees to form a favorable psychological state and behavioral performance (Zhang et al., 2018). Moreover, research shows that the formation of *moqi* between subordinates and supervisors requires the subordinates to accurately interpret the information provided by their supervisors (Zheng et al., 2017b). Whether subordinates can or cannot interpret it completely and properly may be affected by their ability of perspective taking.

As a trait of individual difference, perspective taking can be defined as an individual’s tendency to spontaneously understand and adopt the psychological point of view of others (Davis, 1983). Contrary to real life, which focuses on expressing one’s own opinions, perspective taking refers to the ability to try to understand the world from the perspective of others (Galinsky et al., 2005), imagine or speculate on others’ opinions and attitudes, and perform cognitive and emotional reactions (Parker et al., 2008). Existing studies have found that, on the one hand, perspective taking can increase the individual’s understanding of others’ emotions, thereby increasing the possibility of individuals to make empathic responses to others (Vaish et al., 2009). On the other hand, perspective taking can increase the overlap degree between the self and others, that is, when adopting someone’s opinion, an individual will perceive that he has more similarities with the object selected by the opinion. Moreover, the information representation of the self and others will merge psychologically (Goldstein and Cialdini, 2007), and an individual’s evaluation of the self and others will also change accordingly (Galinsky et al., 2008).

In an organization led by a high level of AL, individuals show their inner thoughts, opinions, and feelings to others (Kernis and Goldman, 2006; Wood et al., 2008). Therefore, if a leader likely shows authenticity and credibility, the subordinates will show their own unique thoughts and emotions. Showing the truth together is conducive to the formation of *moqi*. The effect of AL on the formation of subordinates’ moqi depends on the degree to which subordinates listen to the views of their leaders and integrate them into their own thoughts. That is, the subordinates’ high-level of perspective taking is in favor of an in-depth understanding of these effects. First, Goldstein and Cialdini found that when adopting someone’s point of view, an individual will perceive that he has more similarities with the object adopted. The information representations of the self and others will merge psychologically, accepting and understanding others’ ideas and resources (Goldstein and Cialdini, 2007), that is, subordinates with a high-level of perspective taking will think that they have more similarities with their leaders. In addition, they will look at problems from the perspective of the leaders, have a deeper understanding of the role model of authentic leaders, and accurately understand and analyze the work of the leaders’ expectations, requirements, and behavioral intentions, which is beneficial to the cultivation of subordinates’ *moqi.* Second, employees with a high-level of perspective taking will invest in more cognitive resources (Gilin et al., 2013) to interpret the information provided by the leaders as objectively as possible, which promotes the formation of *moqi* between subordinates and leaders. In addition, when trying to understand other’s point of view, subordinates will be prompted to enter deeper and more complex ways of thinking. In turn, they process information more thoughtfully, thoroughly, and effectively (Gruenfeld, 1995; Gruenfeld et al., 1998).

While leaders have a high level of AL, the subordinate with a low level of perspective taking cannot accurately understand, actively adopt, and move forward what the leaders express. More specifically, when the subordinates have a low level of perspective taking, the feedback of the leaders can be easily interpreted from their own perspective, which is very subjective and causes a cognitive bias (Yaniv and Choshen, 2012). They may be unwilling to invest in enough cognitive resources to understand the intentions or ideas of their leaders (Parker et al., 2008) and require a little understanding of the expectations of their leaders. Hence, ineffective communication or misinterpreting information may exist between the leaders and subordinates, which is not conducive to the cultivation of *moqi* between them (Parker et al., 2008; Wang et al., 2018). Thus, this study proposes the following hypothesis:

Hypothesis 4: *Perspective taking positively moderates the relationship between AL and subordinates’ moqi, such that the relationship is strongest when perspective taking is higher.*

Integrating the relevant discussions of Hypothesis 3 and Hypothesis 4, this study further predicts that perspective taking will moderate the indirect effect of subordinates’ moqi between AL and employee’s taking charge behavior, that is, the moderated mediating role. Specifically, when employees have a high level of perspective taking, they are more likely to accept and understand the traits and behaviors of authentic leaders. Meanwhile, they will be more identifiable with the process and results of decisions made by the leaders, thereby enhancing subordinates’ moqi with the leaders, which not only reduces the uncertainty in the interaction between subordinates and supervisors, but also helps the subordinates obtain more information and support from their supervisors, and then, promote employee’s taking charge behavior. Conversely, when employees’ perspective taking is low, they prefer to interpret the leader’s behavior from their own perspective, which is not conducive to the cultivation of subordinates’ moqi with leaders. At this point, they are more likely to split in an interactive process, which will make the employees’ taking charge activities lack the necessary resource support, thereby weakening employee’s taking charge behavior. In summary, the following hypothesis is proposed:

Hypothesis 5: *Perspective taking moderates the indirect effect of subordinates’ moqi on the relationship between AL and employee’s taking charge behavior, such that the indirect effect is stronger when employees’ perspective taking is high.*

**FIGURE 1:**
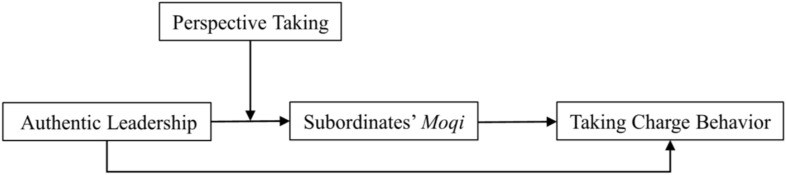
Theoretical model.

Figure 1 depicts our theoretical framework.

## Materials and Methods

### Sample and Procedures

The questionnaire data were collected from several enterprises in Lanzhou, Harbin, Tianjin, and Nanjing in China. Data were collected by the supervisor–subordinate pairing mode and multiple sources to minimize a common method bias (Podsakoff et al., 2003). The survey was conducted in two phases, commencing in February 2020 and ending in May 2020. At phase 1, we distributed questionnaires to 450 employees to survey their evaluation of AL, perspective taking, and demographics information. Among them, 406 valid questionnaires were collected. Three months later, at phase 2, we surveyed the 406 respondents on *Moqi* with their direct supervisors and asked the respondents’ direct supervisors to evaluate employee’s taking charge behavior. The questionnaire was filled in anonymously, and the number was set to complete the two-phase matching. The completed questionnaire was sealed and collected by specialized personnel on the spot.

The survey initially involved 85 supervisors and 450 subordinates. The invalid ones were deleted, and 323 matched supervisor–subordinate dyads from 323 employees and 70 supervisors were finally collected. On average, each supervisor provided ratings for 4.6 employees. In terms of demographic statistics, 56% of the respondents were males. A majority of the respondents were 25–35 years old (85.45%). Of the respondents, 47 (14.55%) had the highest educational qualification of junior college or below, 170 (52.63%) had a bachelor’s degree, 96 (29.41%) had a master’s degree, and the remaining 10 (3.1%) had completed a doctorate. The average organizational job tenure of the employees was 25 months.

### Measurement

Because the original instrument was developed in English, we translated the survey into Chinese using the standard method of translation and back-translation to ensure comparability (Brislin, 1970), and some items were adjusted to the research context. All participants responded on a six-point Likert-type scale, ranging from 1 “totally disagree” to 5 “completely agree.”

**Authentic leadership.** Employees rated their authentic leaders according to Walunmbaw et al.’s 16-item AL measure (2008), which includes specific items, such as “My supervisor can clearly know the impact of some specific behaviors on others” and “My supervisor encourages subordinates to express their values independently.” The internal consistency Cronbach’s α coefficient in this study was 0.943.

**Subordinates’ *Moqi*.** Zheng et al. (2017b) 8-item scale was used to measure subordinates’ *moqi* with their direct supervisor at work. Employees rated the items, such as “I can usually understand any ambiguities and concerns about work for my supervisor” and “I can understand the working methods of my supervisor.” The internal consistency reliability coefficient of the scale was 0.884.

**Perspective taking.** Employees rated their own perspective taking using eight items that were developed and used by Davis (1983). The representative items included “Before criticizing someone, I try to imagine how I would feel if I stood in his/her shoes” and “I believe that there are two sides to every question and try to look at them both.” The internal consistency reliability coefficient of the scale was 0.882.

**Taking charge behavior.** From previous studies, we asked the supervisors to rate subordinates’ taking charge behavior based on the Morrison and Phelps’s 10-item scale (1999). Sample items were “This person often tries to change how his or her job is executed in order to be more effective” and “This person often tries to change organizational rules or policies that are non-productive or counterproductive.” The internal consistency reliability coefficient of the scale was 0.846.

**Control variables.** We controlled some variables that may be related to employee’s taking charge behavior, including gender, age, education, and job tenure (Dysvik et al., 2016; Zheng et al., 2017a). Gender was a dummy coded as 1 = female, 0 = male.

## Results

### Descriptive Statistics and Correlations

Table 1 presents the means, standard deviations, and correlations of all variables in this study. As shown in Table 1, AL is positively correlated with subordinates’ moqi (*r* = 0.336, *p* < 0.01) and employees’ taking charge behavior (*r* = 0.161, *p* < 0.01). Subordinates’ moqi is positively correlated with employee’s taking charge behavior (*r* = 0.156, *p* < 0.01); thus, the relevant hypotheses were preliminarily verified.

**TABLE 1:** Means, standard deviations, and correlations among key variables.

Variable	1	2	3	4	5	6	7	8
1. Gender	–							
2. Age	–	–						
3. Education	−0.072	0.042	–					
4. Job tenure	0.068	0.292**	−0.217**	–				
5. Authentic Leadership	0.063	−0.097	0.042	0.084				
6. Subordinates’ *Moqi*	0.048	−0.119*	−0.157**	0.286**	0.336**			
7. Perspective Taking	0.031	−0.232**	0.002	0.167**	0.366**	0.432**		
8. Taking charge Behavior	−0.019	−0.077	0.012	−0.040	0.161**	0.156**	0.174**	
Mean	1.440	2.100	3.210	2.120	4.771	4.135	4.467	4.489
SD	0.497	0.427	0.723	0.997	0.756	0.884	0.827	0.682

*p < 0.05; **p < 0.01.

### Confirmatory Factor Analyses

Table 2 reports the results of the confirmatory factor analyses (CFA) and Chi-square difference tests. As shown in Table 2, the CFA results indicate that our hypothesized four-factor model (authentic leadership, *moqi*, perspective taking, taking charge behavior) provides a better fit to the data (χ^2^/df = 1.937; CFI = 0.915; TLI = 0.907; RMSEA = 0.054; SRMR = 0.059) than other parsimonious models given that the Chi-square difference test results are all significant at the 0.001 level (Fornell and Larcker, 1981). Based on this analysis, the discriminant validity of the four key variables is good enough to perform further regression analysis.

**TABLE 2:** CFA and Chi-square difference test results.

Model	χ^2^	*df*	χ^2^/df	RMSEA	TLI	CFI	SRMR
Four-factor	1,532.276	791	1.937	0.054	0.907	0.915	0.059
Three-factor	2,629.686	794	3.312	0.085	0.771	0.789	0.101
Two-factor	3,814.773	796	4.792	0.108	0.624	0.652	0.120
One-factor	5,219.469	814	6.412	0.129	0.463	0.493	0.142

***P < 0.001. AL, authentic leadership, MQ, Moqi, PT, perspective taking, TCB, taking charge behavior. Four-factor: AL, MQ, PT, TCB; Three-factor: AL + MQ, PT, TCB; Two-factor: AL + MQ + PT, TCB; One-factor: AL + MQ + PT + TCB.

### Hypotheses Testing

**The relationship between AL and taking charge behavior**. Hypothesis 1 posits that a significantly positive relationship exists between AL and employee’s taking charge behavior. As reported in Table 3 and in line with Hypothesis 1, the results show that AL is positively related to the taking charge behavior (β = 0.161, *p* < 0.01), thereby supporting Hypothesis 1.

**TABLE 3:** Hierarchical regression results.

Variable	Subordinates’ *Moqi*				Taking charge behavior			
	Model 1	Model 2	Model 3	Model 4	Model 5	Model 6	Model 7	Model 8
Gender	0.017	0.000	0.001	0.000	−0.019	−0.028	−0.028	−0.027
Age	−0.212***	−0.170	−0.089	−0.098	−0.074	−0.051	−0.028	−0.013
Education	−0.075	−0.100	−0.115*	−0.111*	0.010	−0.003	0.011	−0.010
Job tenure	0.331***	0.289***	0.220***	0.213***	−0.015	−0.037	−0.077	−0.070
Authentic leadership		0.299***	0.203***	0.219***		0.161**	0.120*	0.116*
Subordinates’ *Moqi*							0.137*	
Perspective taking			0.301***	0.321***				0.141*
Authentic leadership * Perspective taking				0.106*				
R^2^	0.132***	0.219***	0.290***	0.300*	0.007	0.032**	0.047*	0.047**
△R^2^	0.132***	0.087***	0.157***	0.010*	0.007	0.025**	0.015*	0.041**

*p < 0.05; **p < 0.01; ***P < 0.001.

**The mediating effect of subordinates’ *moqi*.** We followed the suggestion of Baron and Kenny (1986) to test whether the following three conditions for mediation effect were satisfied to test if subordinates’ *moqi* would serve as a mediator between AL and taking charge behavior: (1) The independent variable should have a significant relationship with the dependent variable; (2) The independent variable should be significantly associated with the mediator; (3) After controlling for the independent variable, the mediator should be significantly related to the dependent variable and the coefficient of the effect of the independent variable on the dependent variable becomes weaker (a partial mediation) or non-significant (a complete mediation).

Specifically, given that Hypothesis 1 is supported, the first condition is satisfied. In Model 2, AL is significantly positively related to subordinates’ *moqi* (β = 0.299, *p* < 0.001); thus, Hypothesis 2 is verified, thereby satisfying the second condition. In Model 7, after controlling for AL, the coefficient of subordinates’ *moqi* on employee’s taking charge behavior is significant (β = 0.137, *p* < 0.05). Furthermore, the relationship between AL and employee’s taking charge behavior becomes weaker (from β = 0.161, *p* < 0.01 in Model 6 to β = 0.120, *p* < 0.05 in Model 7). Hence, the third condition is also met. Combining these three conditions, subordinates’ *moqi* partially mediates the relationship between AL and employee’s taking charge behavior, thereby supporting Hypothesis 3.

**FIGURE 2:**
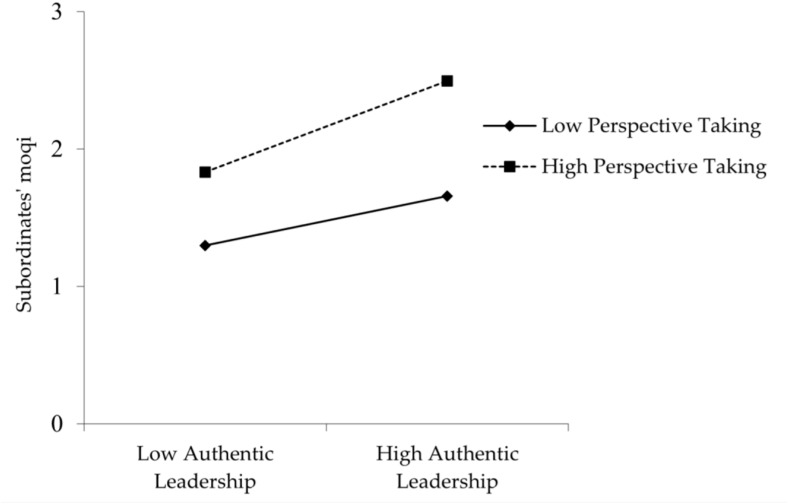
The moderating effect of perspective taking on the relationship between authentic leadership and subordinates’ *moqi.*

**The moderating effect of perspective taking.** Hypothesis 4 predicts that the indirect effect of subordinates’ *moqi* in the relationship between AL and taking charge behavior will be strengthened by a high level of perspective taking. This study first standardized the independent and moderated variables separately to eliminate the influence of collinearity on the research results. Then, the standardized values were multiplied to further carry out the hierarchical regression analysis. Table 3 shows the results for this. The interaction term (authentic leadership × perspective taking) on subordinates’ *moqi* is positively significant (β = 0.106, *p* < 0.05; Model 4 in Table 3); thus, Hypothesis 4 is supported. As suggested by Cohen et al. (2013), we construct the interaction diagram of the moderation effect under different levels of perspective taking to greatly understand the form of the interaction. As depicted in Figure 2, AL is more strongly related to subordinates’ *moqi* when perspective taking is higher rather than lower, which further verifies Hypothesis 4.

**The moderated mediating effect test.** Finally, we analyze Hypothesis 5 according to the suggestion of Preacher et al. (2007). In this study, we adopt Mplus7.0 to simulate the parameters bootstrap for 20,000 times to test the mediated moderation model in the context of one standard deviation above and below the mean. As reported in Table 4, under the 95% confidence interval, the indirect effect of authentic leadership on employee’s taking charge behavior through subordinates’ moqi is not significant in a low perspective taking, while the effect value of a high perspective taking is significant, and the difference between the two groups is also significant. Meanwhile, the difference value of the indirect effect at a different perspective taking level does not contain zero in 95% CI, therefore, Hypothesis 5 is verified.

**TABLE 4:** Moderated mediating effect test.

Mediating effect: AL→MQ→TCB				
Moderator: PT	Indirect effect	Standard error	95% Confidence interval of indirect effect	
			Low	High
High PT (+*SD*)	0.019	0.011	0.008	0.045
Low PT (−*SD*)	0.028	0.065	−0.027	0.059
Indirect effect difference	0.036	0.019	0.022	0.076

*AL, authentic leadership, MQ, Moqi, PT, perspective taking, TCB, taking charge behavior.*

## Discussion

This research aims at investigating the internal mechanism and boundary condition between authentic leadership and employee’s taking charge behavior. Specifically, the findings of this research indicate that AL enhances employee’s taking charge behavior, and that subordinates’ moqi plays a partial mediating role in the above relationship. In addition, employees’ perspective taking positively moderates the positive relationship between AL and subordinates’ moqi as well as the mediating effect of subordinates’ moqi in the relationship between AL and employee’s taking charge behavior. The following sections will focus on the theoretical and practical implications of this research, discuss the limitations of our work, and propose future research directions.

### Theoretical Implications

This research enriches the existing literature concerning authentic leadership and employee’s taking charge behavior, and makes several theoretical implications. First, this paper reveals the positive effects of AL on employee’s taking charge behavior. It expands the research on the influence of AL as a new type of leadership behavior. At present, many scholars have explored the influence of AL on the employees’ innovative, organizational citizenship, voice, and other positive work behaviors, without paying enough attention to the taking charge behavior (Searle and Barbuto, 2013; Zduran and Tanova, 2017). Taking charge emphasizes change and improvement of the current situation, which is of great significance to the development of organizations (Bindl and Parker, 2010). Additionally, it enriches the theoretical research on the antecedents of the taking charge behavior from the leadership aspect.

Second, this paper reveals why AL promotes employee’s taking charge behavior from the perspective of the role identity theory. The study explores the mechanism of subordinates’ *moqi* in the above relationship and finds that the unique personal characteristics of AL lead an effective “action roadmap” for employees to reach a *moqi* state, thereby promoting employee’s taking charge behavior. Although numerous studies have explored the influence of AL on the employees’ proactive work behavior, the majority are from the perspective of the leader-member exchange theory, which cannot fully reflect the characteristics and essence of AL (Lin and Zhao, 2016). The theory of role identity emphasizes the role behavior in accordance with the established role norms (Grant et al., 2011). It shows that the authentic leaders transmit individual role characteristics to their employees through an open interpersonal relationship, and promote the *moqi* state between the two parties, thus, showing the taking charge behavior. Accordingly, this study takes the role identity theory as the framework and introduces subordinates’ *moqi* as the action path, providing a more appropriate perspective for understanding the nature of AL and its influence on employee’s taking charge behavior.

Third, this study enriches the boundary condition between AL and employee’s taking charge behavior, which determines the effect of the above relationship. The process of an individual’s role identity is also affected by his/her own factors. This study incorporates perspective taking into the research framework, which is more conducive to comprehensively reflect the influence of leadership style on the employees’ behavior. In this study, compared with the low levels of perspective taking, employees with high levels of perspective taking will have more *moqi* with their supervisors when they are guided and encouraged by AL. This finding expands our understanding of the boundary conditions for the effectiveness of AL.

### Practical Implications

In addition to the theoretical implications noted above, our results also have implications for practice. First, the research results show that AL is conducive to stimulating employee’s taking charge behavior. Therefore, the organization should encourage leaders to adopt an AL style, guide and train employees by showing their true self, encouraging, participating in decision-making, taking responsibility, and sharing information, so as to reduce the resistance and risk of employees’ taking charge. As for the organization, given that the original leadership style of each manager is diverse, the organization should select the new managers’ ability and trait tendency based on actual needs, thus reducing the resistance for employees to take charge actively. Moreover, it is necessary to conduct AL courses such as theoretical learning and practical role-playing training for the in-service management.

Second, since subordinates’ *moqi* can promote their taking charge behavior, the organization should look squarely at the positive role of subordinates’ *moqi* with supervisors, take the initiative to get along, and improve the degree of *moqi*, thus stimulating the enthusiasm of individuals to take charge. Accordingly, the organization should take effective measures, such as building a platform outside of work to enhance the communication between subordinates and supervisors, so that the subordinates can timely understand the leader’s work objectives and requirements, enhance the sense of identity between them, which further contribute to ensuring that they can effectively release value.

Third, high levels of perspective taking will enhance the cultivation of AL on subordinates’ *moqi*, as well as the whole mediating effect, which reminds enterprises to pay attention to the inspection and cultivation of employees’ perspective taking. More specifically, on the one hand, enterprises can evaluate the candidates’ perspective taking ability by means of group discussion, role playing, or questionnaire measurement, and give priority to those with a strong perspective taking ability in the recruitment and selection process. On the other hand, for those employees who have already joined the company, the organization should attach importance to cultivate and improve their ability of perspective taking. For example, managers can guide employees to think from multiple perspectives and communicate more often with colleagues in their daily work. Enterprises can also organize related activities and trainings frequently to strengthen the employees’ perspective taking ability consciously.

### Limitations and Future Research

This research has several limitations, which suggest meaningful future research directions. First, although this study adopts a longitudinal study of two phases, subordinates’ *moqi* (mediating variable) and employee’s taking charge behavior (dependent variable) in the overall model are collected at the same time point, which is not conducive to clarify the causal relationship between them. In future research, we should use three time points or experimental methods to further infer the causal relationship between variables. Next, even though this study confirms the mediating role of subordinates’ *moqi* in the relationship between AL and employee’s taking charge behavior based on the perspective of the role identity theory, the included control variables were too few to exclude the other possible mechanisms, such as the individuals’ initiative personality (Huang and Yu, 2019) and leader-member exchange process (Hagen and Aguilar, 2012). Future research can control other mechanisms that may affect employee’s taking charge behavior, so as to enhance the reliability of research conclusions. In addition, our research uses *moqi* as our mediation “key” that is believed to be a particularly salient construct in certain Eastern contexts (Barkema et al., 2015), however, due to the influence of cultural factors, whether the conclusions obtained in this study are or are not applicable to the Western organizational environment needs further testing. What is more, due to the limitation of the conditions, this study only selects some enterprises in Lanzhou, Harbin, and other places. In the future, the sample range should be expanded to improve the universality of the research conclusions.

## Data Availability Statement

The raw data supporting the conclusions of this article will be made available by the authors, without undue reservation.

## Ethics Statement

Ethical review and approval was not required for the study on human participants in accordance with the local legislation and institutional requirements. Written informed consent for participation was not required for this study in accordance with the national legislation and the institutional requirements.

## Author Contributions

QW conceived the theoretical model and organized the data collection and analysis. QW and RL edited the manuscript and improved the flow of the manuscript. JL supervised this study and made valuable suggestions for both the initial draft and subsequent revisions. All authors contributed to the article and approved the submitted version.

## Conflict of Interest

The authors declare that the research was conducted in the absence of any commercial or financial relationships that could be construed as a potential conflict of interest.
